# Kernel-based dynamic ensemble approach for classifying imbalanced data with overlapping classes

**DOI:** 10.1038/s41598-026-42940-y

**Published:** 2026-04-30

**Authors:** Somiya Abokadr, Azreen Azman, Hazlina Hamdan, Nurul Amelina Nasharuddin

**Affiliations:** 1 Present Address: Faculty of Computer Science and Information Technology, Universiti Putra Malaysia, Serdang, 43400 Selangor Malaysia; 2 Faculty of Science, University of Zintan, Al Zintan, Libya

**Keywords:** Imbalanced data, Dynamic ensemble, Kernel method, Classification, Overlapping classes, Engineering, Mathematics and computing

## Abstract

In many real-world applications, binary and multi-class classification problems involving imbalanced data and overlapping boundaries present a significant challenge for traditional machine learning algorithms. In this paper we propose a Dynamic Ensemble Selection framework using a Boundary-Aware Kernel (DES-BAK). We investigate the use of ensemble learning approaches to tackle this problem. The aim is to enhance classification tasks based on accuracy, precision, and G-mean by proposing an ensemble of classifiers that leverage different feature representations and classification algorithms. We introduce a novel boundary separation method for the kernel function to separate the imbalanced classes, reduce overlapping, and further improve the ensemble’s performance. The purpose of this method is to divide overlapping boundaries in the classification process. We assess the efficacy of the given method within the framework of imbalanced data in binary and multi-class skewed classification issues with overlapping constraints through Experiments conducted on 15 benchmark datasets. The results demonstrate that the framework surpasses several state-of-the-art methods in terms of classification accuracy. The combination of diverse feature representations, classification algorithms, and the innovative boundary separation approach enhances the ability of the ensemble to handle imbalanced data and overlapping boundaries. These findings showcase the potential of proposed an approach in addressing challenging classification scenarios and contribute to advancing machine learning techniques in real-world applications.

## Introduction

Class imbalance is a major problem in the field of machine learning and data mining. In imbalanced datasets, a single class has more samples in it, whereas the other one is usually the more important one and therefore represented with fewer samples. This imbalance can lead to serious issues, rendering many carefully designed machine-learning systems ineffective. Traditionally, classifiers aim to minimize overall classification error. However, when learning from imbalanced data, classifiers tend to make more errors on minority class examples compared to majority class examples^1^. The prevalence of class imbalance is particularly pronounced in real-world scenarios, such as fraud detection, spam filtering, software defect prediction, and more^2^.

Also, there is too much overlap of classes to make it even worse. Class overlap. These are instances of the same class that overlap in the data space. The similar feature values in these instances are in different classes. Such issues like the detection of electronic fraud during transactions are very challenging, as it is a problem with both class imbalance and overlap^3^. Recent research proves that, although class imbalance on its own reduces the performance of a classifier, there is a further overall increase in minority-class errors through overlapping class distributions (Prati et al., 2004^4^; He and Garcia, 2009^5^; Fernández et al., 2018)^6^. This compounding effect is especially troublesome with such tasks as fraud detection and anomaly detection, where the minority-class examples are not only rare but also expensive to misclassify (López et al., 2013^7^; Johnson and Khoshgoftaar, 2019)^8^.

In this context, the limitations of existing methods, for handling class imbalance fall into several categories:

**Data-Level Methods**: The distribution of class instances is changed by these techniques. Oversampling the minority class or undersampling the majority class are common tactics. Nevertheless, data-level approaches might worsen overfitting and information loss, which would impact model performance overall^9^.
**Algorithm-Level Methods**: These approaches modify the learning algorithm itself. For instance, they may assign more weight to misclassified minority class instances during training. While effective, changes to the algorithm may not always be user-friendly^10^.
**Hybrid Methods**: Hybrid approaches mix data-level and algorithm-level techniques. The objective is to strike a balance between maintaining model efficiency and addressing class imbalance.
**Cost-Sensitive Learning**: is used when various misclassification costs are placed on various classes. Nevertheless, it is difficult to choose relevant cost ratios.
**Deep Learning**: Deep learning models have shown promise in handling imbalanced data. However, they also face challenges related to class overlap and the complex structure of large datasets^11^.

### General problem

**Fig. 1:**
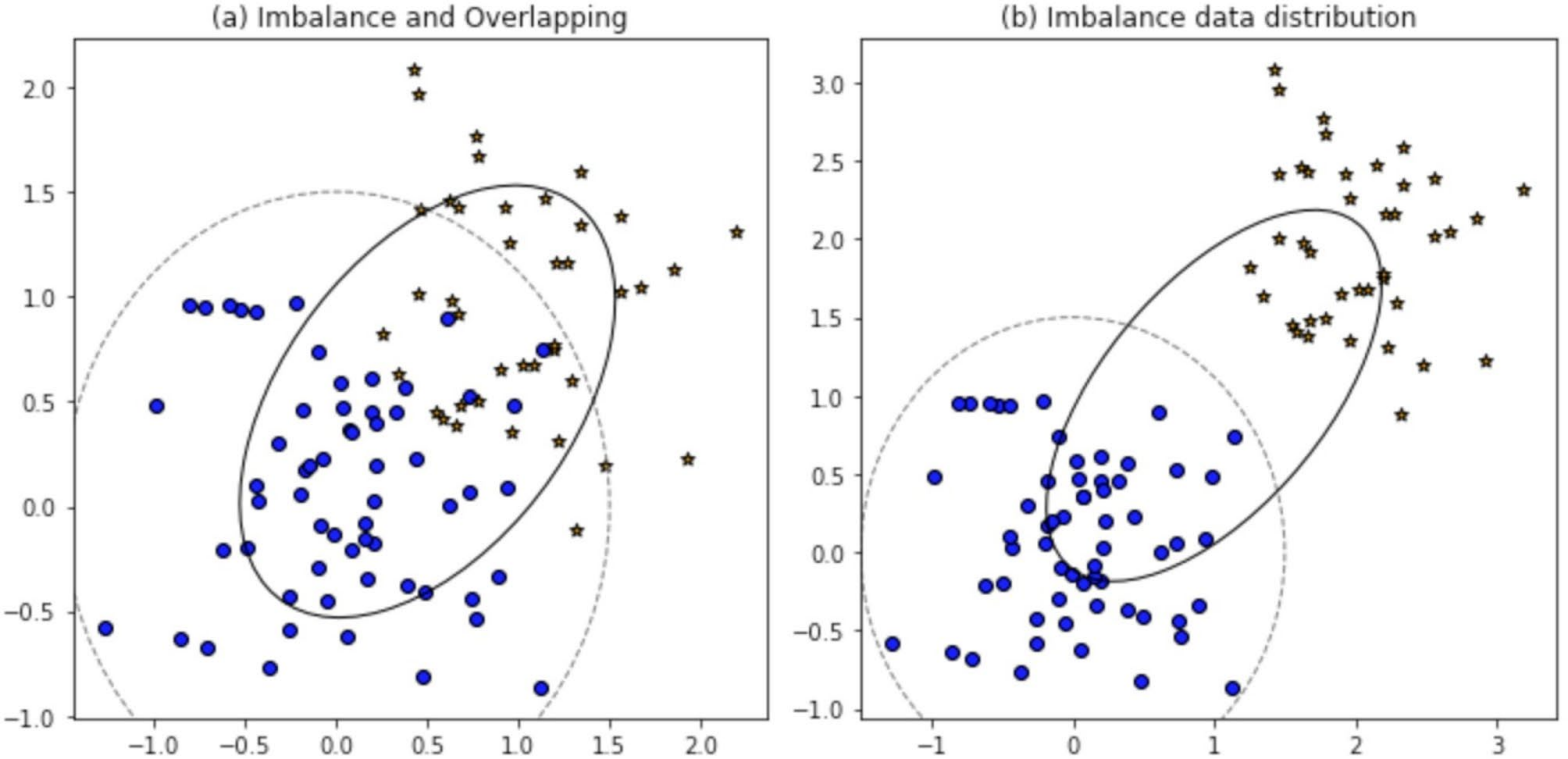
Imbalance, Overlapping, and Distribution of Datapoints: (a) Data Imbalance and Overlapping, (b) Imbalance Data Distribution.

The use of big data and Internet of Things (IoT) applications creates large amounts of high-dimensional data, which cover a wide range of domains, including healthcare, finance, security, and decision support systems^12,13^. Classification approaches based on machine learning have become common to uncover valuable patterns in such data and aid the predictive decision-making^14,15^. Nevertheless, one of the inherent problems in numerous real-world classification problems is class imbalance, in which a class (minority classes) is drastically underrepresented relative to other classes (majority classes). This disparity considerably lowers the learning performance because most conventional classifiers are meant to maximize the overall accuracy and thus are skewed on majority classes^16,17^. The cases of a minority-class in an unbalanced environment are common to the critical cases (fraud, faults, anomalies), so the cost of their misclassification can be especially high. This becomes more subtle, as the increase in data dimensionality leads to the increase of the difficulty of learning reliable decision boundaries for minority classes because of the lack of sample coverage in the feature space (He and Garcia, 2009^5^; Branco et al., 2016^18^). Though there can be a possibility of overlap of classes in some areas of the feature space, it is not considered as a major issue in this work. Rather, the concept of overlap is to be seen as a secondary effect that may further raise the misclassification of minorities with respect to the classes in which they belong, already unequal datasets. Based on this fact, the central theme of this paper is the efficient processing of the class imbalance of high-dimensional data through the enhancement of the minority-class recognition and the overall model robustness. The demonstrated illustrative representation is only meant to give intuition ideas and is in no way an indication of the actual data distribution in the real world (Fig. 1).

### Specific problem

**Fig. 2:**
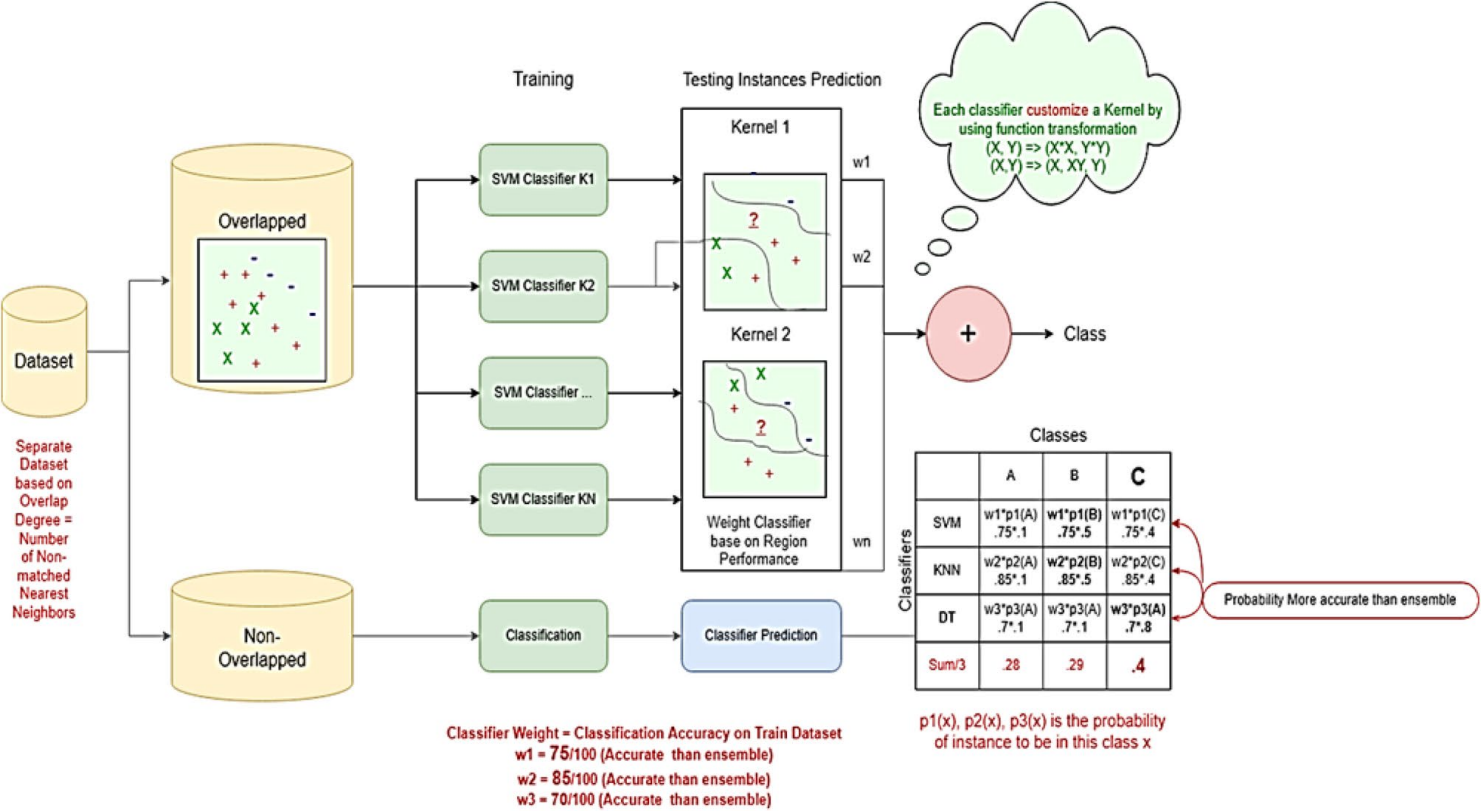
Customised Ensemble Classifier Flow.

**Fig. 3:**
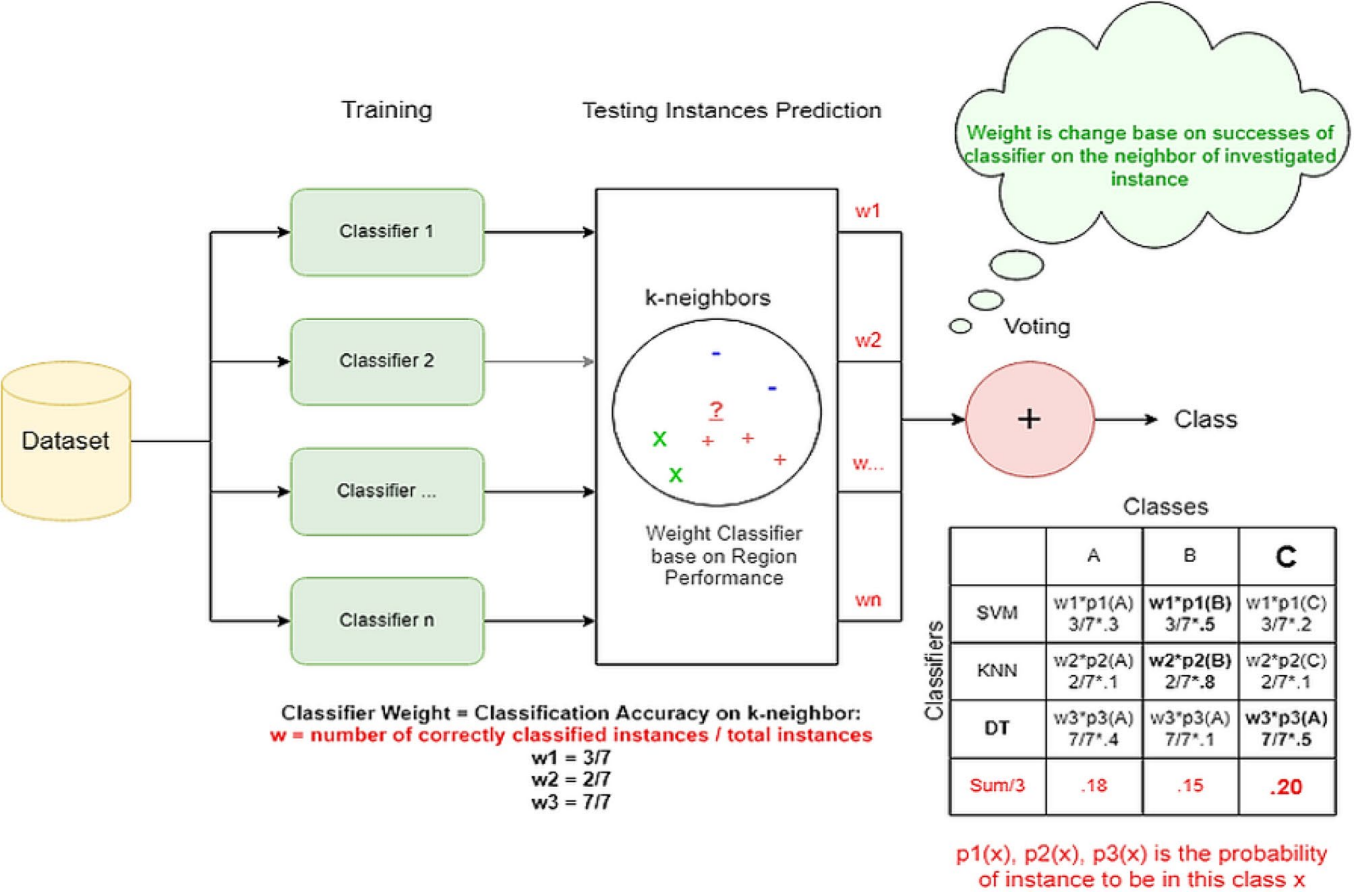
Dynamic ensemble classifier flow.

In the scholarly discourse on addressing challenges posed by multi-class imbalanced and overlapping data, several notable strategies have emerged. These include algorithm adaptation^19^, data-level techniques^20^,hybrid approaches,^21^ and ensemble-based strategies^22^. A. Fernndez, S. Garca, F. Herrera, Addressing the classification with imbalanced data: open problems and new challenges on class distribution, in^23^. Data-level methods typically involve resampling to rebalance the distribution of class samples. Algorithm-level methods, on the other hand, either develop new classifiers or adapt existing ones to better manage imbalanced datasets^24^. Hybrid methods combine elements of both data and algorithm-level techniques, often incorporating ensemble methods to form a comprehensive solution.The key components of the framework as illustrated in the new Figs. 2 and 3, including the balanced training set generation, the customized kernel functions for boundary separation, and the dynamic ensemble selection.

The Support Vector Machine (SVM) algorithm, a supervised learning tool, is widely used for classification tasks. Originally conceived for binary classification, it operates by constructing an optimal hyperplane within an n-dimensional space that best separates the training data into distinct classes^25^. SVMs can be equipped with a variety of kernel functions, the selection of which depends on the specific characteristics of the dataset at hand. However, when deploying SVMs for classification purposes, several critical issues arise, such as choosing the appropriate kernel function, ensuring accuracy, and maintaining efficiency during the training and testing phases. Additionally, it is crucial to determine the correct order and values for various parameters to achieve optimal generalization performance^26^.

Modifications to algorithms are often tailored to specific scenarios, particularly when addressing overlap issues in datasets. The limitations of existing data-level, algorithm-level, hybrid, and ensemble-based methods are now more explicitly articulated, especially with respect to their scalability, generalizability, and sensitivity to kernel selection in high-dimensional imbalanced settings. Furthermore, the motivation for adopting an SVM-based dynamic ensemble framework has been strengthened by clearly identifying the unresolved challenges^10^. As the number of classes increases and more attributes are shared among samples, it becomes increasingly complex for SVMs to delineate an optimal separating hyperplane. This difficulty is one of the primary concerns when adapting SVM algorithms to handle complex datasets^27^. To address these challenges, a novel approach has been proposed^27^. This method involves analyzing the regions where class overlap occurs and utilizing statistical data from these regions to create a suite of kernel functions tailored to each level of overlap. These kernels are then employed by a set of SVM classifiers, which are further enhanced with AdaBoost, a well-known ensemble method for improved classification. The ensemble’s final decision is made through a voting mechanism. Furthermore, AdaBoost’s ensemble framework is traditionally recognized for its efficacy in general classification problems. Building on this foundation, a dynamic ensemble learning classification algorithm has been introduced^28^. This algorithm evaluates the competence of candidate classifiers based on their ability to classify instances within their neighborhood accurately. It is explicitly designed for multi-class imbalanced datasets and aims to outperform the conventional AdaBoost algorithm by dynamically adapting to the unique challenges presented by such data.

### Contribution

To propose a dynamic ensemble learning based on SVM with different kernels, customizing those emphases on overlapped samples for imbalanced data classification problems. The primary reasons for degrading the performance of machine learning algorithms include overlapping and imbalanced datasets, as well as uneven sample distribution. The drawback of an imbalanced class distribution leads to a deterioration in machine learning performance. In such cases, the training strategies of multi-sample selection based on ensemble learning and multi-classification pay more attention to the overlapping class area. Hence, the design framework for establishing class overlap combines. The several difficulties in learning from the multi-class imbalance contribution of this paper are as follows:

- A kernel-based dynamic ensemble algorithm is proposed that concentrates model construction on class overlap regions identified via nearest-neighbor analysis.
- The method employs similarity-weighted SVM training and kernel diversity to select locally optimal classifiers for ambiguous samples.
- An instance-dependent ensemble selection strategy is introduced, where only the most relevant classifiers participate in voting for each test sample.

### Paper structure

The following components make up the primary research article. The existing literature examined in Sects. “Related work” and “Methodology overview” includes a quick overview of the ensemble classifiers. We describe the reduction of overlapping and borderline samples in Sect. “Mechanism”, which discusses the MSVM-operational AdB’s mechanism. Using current techniques in V, we assess the outcomes for the proposed classifier. Section “Conclusion” emphasizes the final remarks.

## Related work

In the field of machine learning, class imbalance and overlapping decision boundaries pose significant challenges for accurate classification. Real-world applications often involve scenarios where one class is significantly more prevalent than another, leading to biased models. Additionally, overlapping regions in feature space make it difficult to distinguish between classes^9^. Imbalanced learning problems refer to situations where the distribution of classes in the dataset is unequal, leading to an over representation of one class and an under representation of the other. This poses a challenge for classification algorithms, as they tend to have lower accuracy for the minority class. This response will provide strategies for handling imbalanced learning problems in binary and multi-class classification tasks based on relevant references.

- **Data Resampling:** Data resampling techniques like Synthetic Minority Over-sampling Technique (SMOTE), Borderline-SMOTE, and Adaptive Synthetic Sampling (ADASYN) have been shown to improve the classification outcomes of an unbalanced dataset^29^. It is a **Algorithm Techniques:** Recent research has shown that deep learning algorithms such as Convolutional Neural Networks (CNNs) and Recurrent Neural Networks (RNNs) can handle imbalanced input. Additionally, to solve class imbalance, conventional techniques like support vector machines (SVMs) and random forests have been updated^30^.
- **Cost-Sensitive Learning:** Cost-sensitive learning approaches like Weighted SVM and Adaptive Cost-Sensitive Boosting have been shown to improve the performance of classifiers on imbalanced datasets^31^.
- **Ensemble Methods:** Ensemble methods, such as bagging, boosting, and stacking, have improved classification performance on imbalanced datasets^32^.
- **Active Learning:** Active learning, which involves selecting the most informative samples for labelling, has been proposed to handle imbalanced datasets. Recent studies have shown that active learning can improve classification performance on imbalanced datasets.

### Critical Analysis of Literature

In machine learning, the challenge of imbalanced data classification stands as a significant hurdle, posing unique difficulties that necessitate advanced solutions. This critical analysis examines various studies that illuminate innovative methodologies and their effectiveness in addressing these challenges, with a particular focus on ensemble learning strategies and their role in improving classification accuracy in datasets characterised by imbalances and overlaps.

Imbalanced datasets frequently occur in real-world situations, where specific classes are significantly underrepresented compared to others. This imbalance leads to biased algorithm performance, often overlooking the crucial minority class, which may hold significant importance in various applications, such as medical diagnoses, fraud detection, or rare event prediction^33^. The imbalance not only skews predictive accuracy but also poses the challenge of class overlap, where classes are not distinctly separable, further complicating classification tasks^34^ One notable advancement in this domain is the Overlap-Sensitive Margin (OSM) classifier, introduced by^35^. This innovation, situated within the fuzzy support vector machine (FSVM) paradigm, augments traditional classification methods by effectively delineating overlapping data into distinct regions. This approach significantly improves classification in datasets with both overlapping and imbalanced features^36^. The OSM classifier combines FSVM with k-nearest neighbours (KNN) to address these challenges, particularly in the multi-class domain. The classifier assigns misclassification costs to FSVM, differing from traditional methods, and uses KNN to measure the degree of overlap for individual samples. This methodology highlights the ingenuity in adapting existing frameworks to better handle the complexities of imbalanced datasets^37^. Another significant contribution comes from^37^, who explore the impact of class overlap, positing it as a more substantial concern compared to class imbalance. Their study stands out for its in-depth experimental approach and critical examination of existing methodologies. They categorize these methods into class distribution-based and class overlap-based strategies, advocating for a research shift towards prioritizing class overlap. This approach highlights the necessity of addressing class overlap to effectively enhance algorithm performance in imbalanced datasets^38^. In addition, the HIO-SVM algorithm, developed by^10^, demonstrates a novel approach to modifying support vector machines (SVMs) for highly imbalanced and overlapping classification scenarios. This method iteratively changes kernel spaces to find all non-overlapping samples in distinct feature spaces, addressing the issue of linear separability in traditional SVMs. This innovative technique ensures accurate prediction of all minority samples while reducing the error rate of majority samples, showcasing a sophisticated adaptation of SVMs for complex data structures. To contextualize the problem, prior research has established that class imbalance and class overlap negatively impact classifier performance, motivating a range of methods including data resampling (e.g., SMOTE and its variants), cost-sensitive learning, and ensemble-based strategies, each aiming to rebalance class distribution or adapt learning to dataset difficulty factors rather than just improving overall accuracy^39–42^.

Further exploring ensemble learning solutions, the study^43^ presents MSVM, a modified SVM that is utilized with the AdaBoost ensemble classifier (MSVM-AdB) as a basis classifier. Using sample contributions, this method separates the multi-class dataset into over/ non-overlapping regions, then filters these into crucial and less critical areas. The MSVM enhances the classifier’s ability to learn in complex datasets by modifying the standard SVM’s kernel mapping function to map overlapping samples in a higher-dimensional space. Using 20 real datasets with different imbalance ratios and levels of overlap, their experimental results show that MSVM-AdB outperforms conventional classifiers.

(García et al., 2018) also contribute to this discussion with their dynamic ensemble selection for multi-class imbalanced datasets (DES-MI). This methodology addresses the class imbalance problem by creating a preprocessing process that uses random balancing to balance the training dataset. The DES-MI approach involves selecting the most suitable classifiers based on weighted cases in proximity, focusing on classifiers that excel at classifying examples in underrepresented regions. In addition, the ELDB approach uses a bag selection technique and a self-reinforcement strategy to obtain a discriminative bag set for multi-instance learning^44^.

The suggested Boundary Aware Kernel (BAK) is distinctly different because it offers a solution to the problem of class overlap by making use of a local, instance-specific kernel adaptation mechanism. The BAK performs a selective modification of the kernel behavior on boundary samples However, unlike cost-sensitive kernel methods, which use uniform class-level penalties, BAK does not compete on a global scale. Unlike adaptive kernel scaling methods that are based on global parameter tuning, BAK integrates competence-based dynamic kernel adjustment with ensemble selection. Furthermore, as opposed to other methods of the boundary oversampling, like Borderline-SMOTE, BAK does not synthesize any data but maximizes new discrimination between boundaries by re-weighting existing samples directly in the SVM kernel formulation.

This strategy highlights the importance of selecting a tailored classifier in multi-class imbalance scenarios, significantly enhancing classification performance.

## Methodology overview

**Fig. 4:**
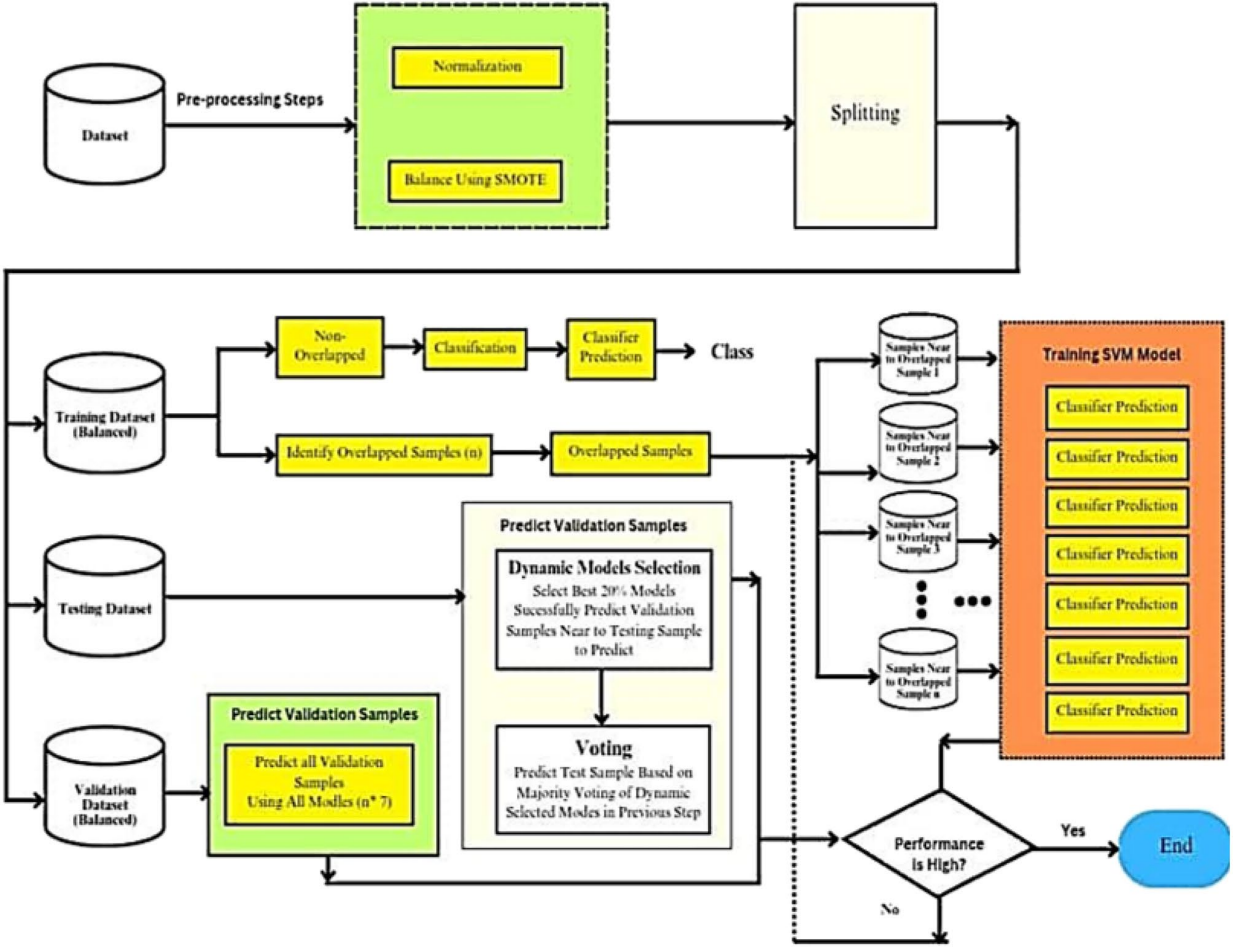
Contribution for Progress Flow Diagram.

The proposed methodology is Dynamic Ensemble Learning for Imbalanced and Overlapping Data. It addresses the challenges of class imbalance and overlapping decision boundaries in classification tasks. Hence, the proposed methodology combines the following key elements:

- **Dynamic Ensemble:** The approach maintains an adaptive ensemble of classifiers (e.g., SVMs) during training. Boundary-Aware Kernel (BAK) or adaptively weighted kernel function, captures data-specific characteristics.
- **Balanced Training Datasets:** We generate balanced datasets using resampling techniques, and classifier selection is data-driven.
- **Preprocessing Steps:** Feature engineering, normalization, and handling missing values are performed as preprocessing steps.
- **Weighting Mechanism:** A weighting mechanism is employed to assign class weights, mitigating the imbalance in the dataset (Fig. 4).

### Framework structure

It encapsulates the key components of the framework, emphasizing its adaptability, focus on data balance, preprocessing steps, and weighting mechanisms to handle imbalanced and overlapping data effectively.

#### Dataset

To examine the methods of the suggested approach, this study selected twenty multi-class imbalanced datasets from the KEEL repository^45^. The selected datasets are described as follows:

- **Automobile.** There are 159 examples in this collection. Twenty-five qualities are used to represent each sample. This dataset’s objective is to categorise a sample as belonging to one of six sorts of the degree scale, indicating that an automobile is riskier than its pricing suggests.
- **Balance scale.** Tip to the right, tip to the left, and balance are the three types of balancing scales that are used in this dataset to model experimental psychology results. Four characteristics-the left weight, the left distance, the right weight, and the appropriate distance-represent each case.
- **Car evaluation.** The hierarchical decision model is the original source of this dataset. Six input qualities (purchasing, main, doors, individuals, lug_boot, safety) and one of four classes (unacceptable, acceptable, reasonable, and very good) are offered for each of the 1728 samples.
- **Heart disease Cleveland**. The V.A. Medical Centre provided this dataset for the detection of cardiac disease. Each case in the five-class classification issue is represented by 14 attributes. The objective is to determine whether the patient has heart disease, ranging from 0 (no disease) to 4.
- **Contraceptive method choice.** The national contraceptive prevalence survey in Indonesia provided this dataset. It forecasts a woman’s preferred method of contraception based on her socioeconomic and demographic traits. Nine characteristics and the method of contraception (no use, long-term, or short-term) are shown for each example.
- **Dermatology.** This dataset comes from an actual dermatological issue. Every example has 34 characteristics. The six forms of dermatology (psoriasis, seborrheic dermatitis, lichen planus, pityriasis rosea, chronic dermatitis, and pityriasis rubra pilaris) can be identified using this method.
- **E. coli**. Each case in this dataset is labelled by one of eight classes and represented by seven attributes. Using measurements of the cell cytoplasm, inner membrane, periplasm, outer membrane, inner membrane lipoprotein, and cleavable signal sequence, this problem seeks to predict the localisation site of proteins.
- **Flare.** There are 1066 samples in this six-class classification issue, each of which is represented by eleven qualities. Predicting a given sample as one of six class labels is the goal of this issue.
- **Glass identification.** The USA Forensic Science Service is the source of this dataset. Each sample is presented with nine qualities. The assignment is to identify one of six types of glass-Na, Fe, K, etc.-that were discovered at the crime scene based on their oxide level.
- **Hayes-Roth.** This simulated dataset was made to examine how prototype-based classifiers behave. Four characteristics-hobby, age, educational attainment, and marital status-represent each example. Since the hobby property was created at random, it is used to introduce noise into the data.
- **LED display domain.** Seven Boolean attributes, one for each light-emitting diode in a seven-segment display, are included in this collection. The task is to find the digit that has been displayed on the screen.
- **Lymphography.** The Institute of Oncology provided this dataset, which is a four-class classification issue. Eighteen attributes and the matching class label are used to represent each sample.
- **New thyroid.** There are 215 samples in this collection, each having three classes and five attributes. Finding out if a patient has hyperthyroidism or hypothyroidism is the issue at hand.
- **Page blocks classification.** Blocks of a document’s page layout that were identified by a segmentation method are included in this collection. Finding the block type text, horizontal line, graphic, vertical line, or picture is the problem at hand.
- **Shuttle.** Nine attributes, all of which are numerical, are used to represent each case in this collection. The task of this dataset is to determine which of seven potential classes-Rad Flow, Fpv Close, Fpv Open, High, Bypass, Bpv Close, and Bpv Open-should be used to regulate the vessel.
- **Thyroid disease.** There are 720 occurrences in this dataset from the diagnosis of thyroid illness, and each instance is represented by 21 attributes. The goal of this dataset is to identify a patient in one of three classes: normal, hyperthyroid, or hypothyroid.
- **Wine.** The findings of a chemical analysis of wines from three distinct cultivars that were grown in the same area of Italy are included in this collection. This dataset’s objective is to categorize wine varieties based on the amounts of thirteen ingredients.
- **Red wine quality.** Red wine samples were used to build this dataset. Each sample is represented by eleven input attributes; the sensory quality that wine experts assess is the output. This dataset’s objective is to rate the quality of red wine.
- **Yeast.** Predicting the protein’s localization site is the goal of this dataset. Eight characteristics are used to characterize each example, and each cell’s location among 10 potential options is labeled.
- **Zoo.** Predicting the kind of animal from seven predetermined classes is the aim of this dataset. Sixteen properties, the majority of which are Boolean values, are used to represent each sample. Each dataset’s characteristics are shown in Table 1, including the number of samples (#Ex.), the number of attributes (#Atts.), the number of numerical (#Num) and nominal (#Nom) attributes, the number of classes (#Cl.), the distribution of classes (#Dc), and the imbalance ratio (IR), which is the ratio of majority to minority examples. Before the partitions were created, the examples in the datasets Automobile, Cleveland, and Dermatology that had missing values were eliminated. Three times five-fold stratified cross-validation (SCV) was used to get the results^46^. In other words, each dataset was divided into five folds, each of which contained about 20% of the dataset’s instances. The method was evaluated on the current fold after being trained for each fold using the instances found in the remaining folds (80% of the dataset cases). In the imbalanced scenario, a 5-fold SCV is more suitable than a 10-fold SCV for the reasons described in^47^.

In order to examine the algorithms on the related webpage, this study chose twenty binary-class imbalanced datasets from the KEEL repository^45^. Table 1 displays the characteristics of the datasets.

**Table 1:** Overview of the datasets used in the experiments.

**Name**	**Instances**	**Classes**	**Features**	**Imbalance ratio**	**Class distribution**
ecoli1	336	2	7	3.36	Majority: 259, minority: 77
ecoli2	336	2	7	5.46	Majority: 284, minority: 52
ecoli3	336	2	7	8.60	Majority: 301, minority: 35
ecoli4	336	2	7	15.80	Majority: 316, minority: 20
glass0	214	2	9	2.06	Majority: 144, minority: 70
glass2	214	2	9	11.59	Majority: 197, minority: 17
glass4	214	2	9	15.46	Majority: 201, minority: 13
glass6	214	2	9	6.38	Majority: 185, minority: 29
haberman	306	2	3	2.78	Majority: 225, minority: 81
new-thyroid1	215	2	5	5.14	Majority: 180, minority: 35
pima	768	2	8	1.87	Majority: 500, minority: 268
segment0	2308	2	19	6.02	Majority: 1979, minority: 329
vehicle0	846	2	18	3.25	Majority: 647, minority: 199
vehicle1	846	2	18	2.90	Majority: 629, minority: 217
vehicle3	846	2	18	2.99	Majority: 634, minority: 212
vowel0	988	2	13	9.98	Majority: 898, minority: 90
Wisconsin	683	2	9	1.86	Majority: 444, minority: 239
yeast1-7	459	2	7	14.30	Majority: 429, minority: 30
yeast2-4	514	2	8	9.08	Majority: 463, minority: 51
yeast3	1484	2	8	8.10	Majority: 1321, minority: 163

#### Preprocessing

In the preprocessing stage of the kernel-based dynamic ensemble approach, two crucial steps are undertaken: normalization and balancing. Normalization Techniques: The document describes the application of min-max normalization, a standard technique in data preprocessing. This method scales all numeric variables in the dataset to a common scale without distorting differences in the range of values. Mathematically, it is represented as (1):

1 $$\begin{aligned} X^{\prime }=\frac{X-X \min }{X \max -X \min } \end{aligned}$$

where *X* is the initial value, $X^{\prime }$ is the normalized value, and $X\min$ and $X\min$ are the variables’ minimum and maximum values, respectively. By ensuring that every characteristic contributes equally to the analysis, this step helps to avoid bias caused by varying scales. Balancing with SMOTE: The Synthetic Minority Over-Sampling Technique (SMOTE) is used to address the challenge of imbalanced datasets. It generates synthetic examples for the minority class, thereby balancing the class distribution. This is crucial for ensuring that the classifier treats each class equally and does not develop a bias towards the majority class. These preprocessing steps are fundamental in setting the stage for accurate and equitable machine learning classification, particularly when dealing with imbalanced datasets that have overlapping classes. The normalization ensures a level playing field for all features, while SMOTE addresses the imbalance in the class distribution, both of which are critical for the success of the subsequent classification stages.

#### Training

**Fig. 5:**
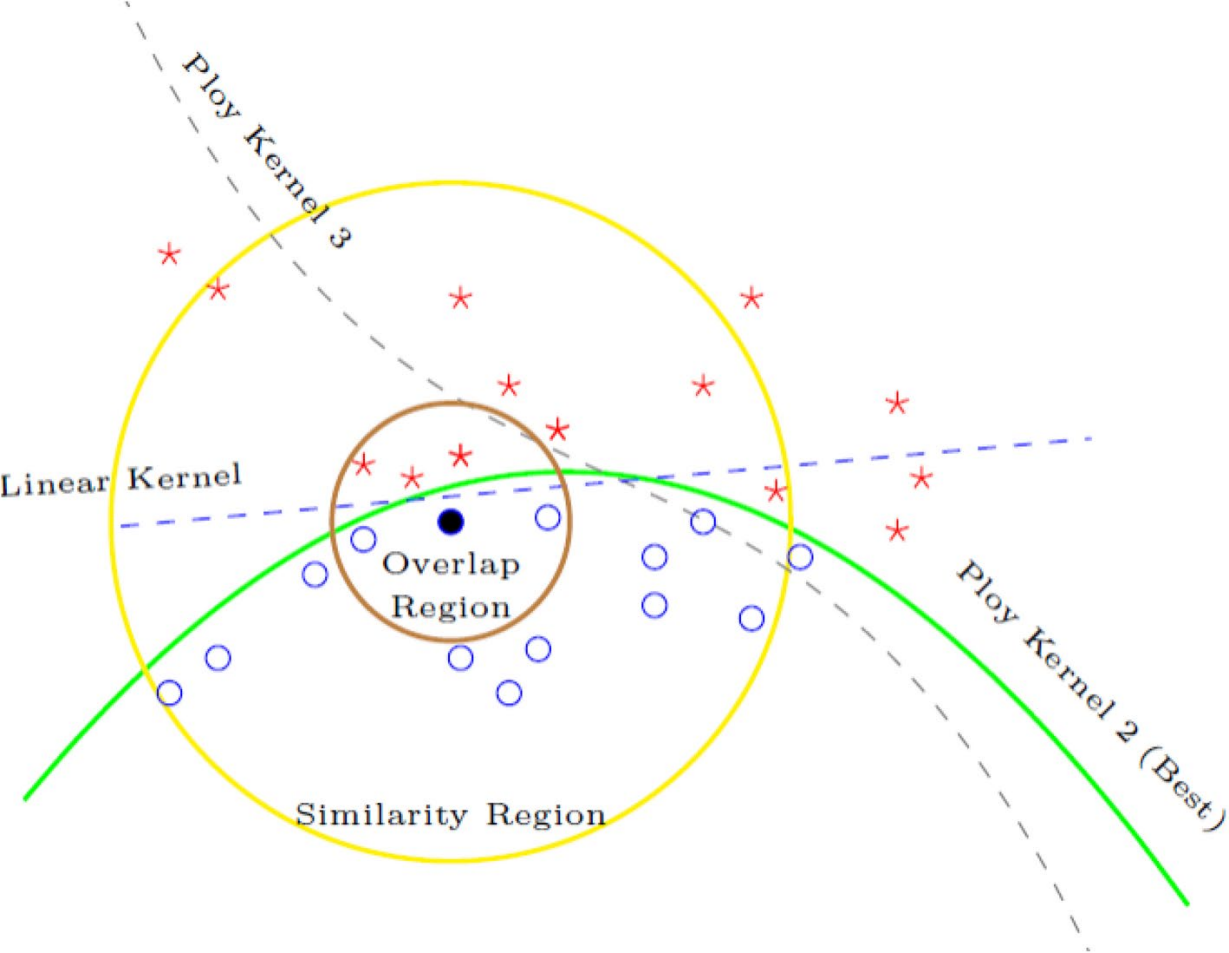
Training.

In the training phase of the kernel-based dynamic ensemble approach, two key steps are performed:

1. The datasets undergo segmentation into three parts: training, validation, and testing. This foundational step ensures that there is a dedicated portion of data for each phase of model development and evaluation.
2. During the sampling phase, we concentrate on separating overlapping samples. This is accomplished by examining the number of nearest neighbors, utilizing the Euclidean distance-a common measure in machine learning. The Euclidean distance is calculated using the formulas in (2) to identify samples that are located in regions with ambiguous class boundaries. The identification of these samples is vital for enhancing classification performance, particularly in areas where different classes overlap or intersect.

2 $$\begin{aligned} { Euclidean Distance }=\sqrt{\sum _{i=1}^n\left( x_i-x_i^{\prime }\right) ^2} \end{aligned}$$

where *x* and $x^{\prime }$ are two data samples *n* is the number of dimensions. This stage involves a more sophisticated process where individualized SVM classifiers, employing a variety of kernels are trained specifically for each sample identified as part of the overlap set, as depicted in Fig. 2. The objective is to select the best model that achieves the highest accuracy. To optimize these classifiers, fine-tuning is performed by adjusting the weights assigned to each training sample. This methodical approach ensures a tailored and precise classification, particularly for samples within the complex overlap set sample (Fig. 5).

#### Prediction

In the prediction phase of the kernel-based dynamic ensemble approach, two critical steps are conducted: classifier evaluation and the implementation of a majority voting process. The research concludes in the fifth stage, which is considered the testing phase, where prediction on the testing dataset takes place. Here, a collective decision-making process is employed, wherein multiple classifiers vote based on their assessed probability of accurately classifying k neighbours from the validation dataset. The classifier ensemble with the highest probability of correct classification influences the final prediction outcome for each test sample. Finally, the system reports the score of accuracy, precision, and G-mean of the proposed classification algorithm. This comprehensive multi-stage approach aims to enhance classification accuracy and reliability in the face of imbalanced data with overlapping class distributions.

## Mechanism

The kernel-based dynamic ensemble approach integrates a meticulous process that begins with the identification and separation of overlapping samples in the sampling phase. This is achieved by evaluating the Euclidean distance equation (2) to pinpoint samples in regions where class boundaries are unclear. The methodology then assigns weights to these identified samples using a similarity measure based on a radial basis function kernel equation (1), as depicted in Fig. 3. In the ensemble training phase, individual SVM classifiers, each employing a variety of kernels, are meticulously trained for samples identified in the overlap set. The approach emphasises fine-tuning the classifiers by adjusting the weights of training samples to better handle the complexities of the overlapping areas. The proposed kernel raises the penalty of the misclassification of the minority-class samples in the overlay areas, biasing the SVM decision border towards the majority one and enhancing the discrimination against minorities. Modification of the standard kernel function $K(\cdot , \cdot )$ as given by definition of the BAK as modified is given by $|human|>$ The BAK is defined formally by modifying the standard kernel function as given by definition as follows.

3 $$\begin{aligned} K_{\textrm{BAK}}(x_i, x_j) = W_i W_j K(x_i, x_j), \end{aligned}$$

In which $W_{i}$ represents the local competence of sample $x_{i}$. The minority-class samples in overlapping regions get higher weights, whereas the majority class samples receive lower weights. This weighting mechanism optimistically adjusts the contribution of every sample to the SVM optimization. The calculation of $W_{i}$ has now been made under the revised Eq. (3).

**Fig. 6:**
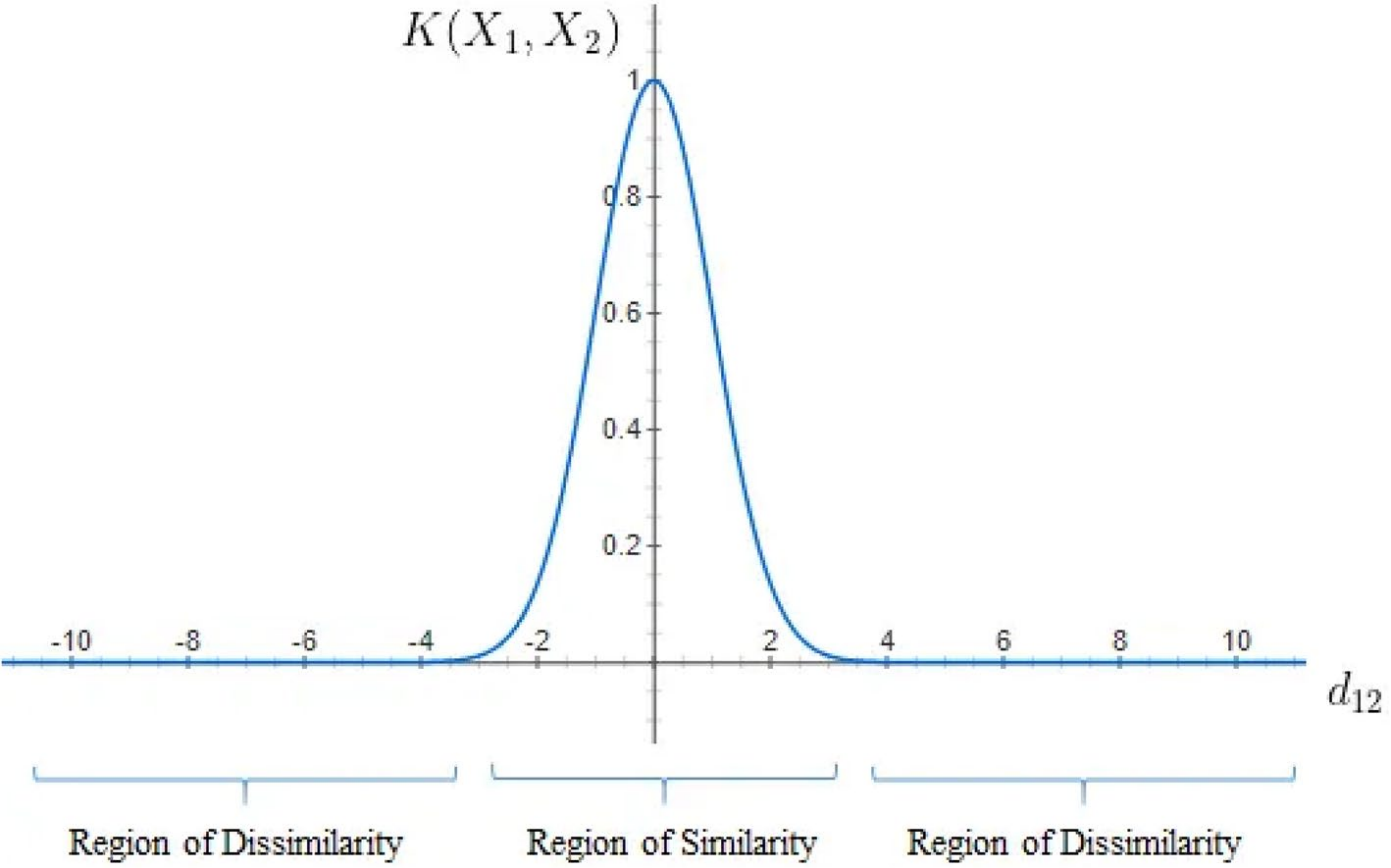
Radial base kernel similarity function.

The testing phase involves a strategic collective decision-making process. Here, classifiers vote on the classification of test samples, drawing on their performance with a selected subset of neighbours from the validation set. The ensemble of classifiers with the most accurate predictions significantly influences the final classification of each test sample (Fig. 6).

The culmination of this process is the reporting of key metrics: accuracy, precision, and G-Mean, offering a comprehensive evaluation of the algorithm’s capability in managing the challenges of imbalanced data with overlapping class distributions. This structured, multi-stage strategy ensures a nuanced, dynamic, and effective approach to classification.

**Algorithm 1:**
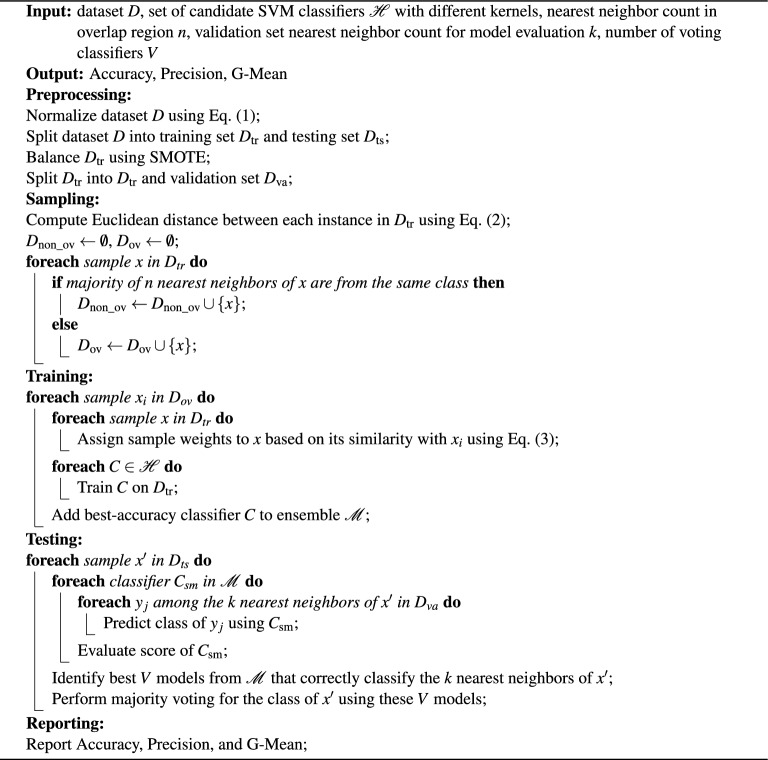
Kernel-based Dynamic Ensemble

## Experimental setup

The experimental settings for the kernel-based dynamic ensemble approach are meticulously designed to optimize classification performance. The experiment employs 10-fold cross-validation to ensure the robustness and generalizability of the results. The data is split into 60% for training, with the remaining 40% equally divided between testing and validation. A diverse array of kernel functions, including linear, polynomial (degrees 2 to 5), sigmoid, and radial base function (rbf), are used to train the SVM classifiers. This variety caters to different data characteristics, enhancing the adaptability of the model. The approach considers four nearest neighbours in the overlapped region and 10 in the validation set for classifier evaluation. Additionally, only the top 20% of classification models are used in the voting process, ensuring that only the most effective models influence the final outcome. The number of nearest neighbours of the minority sample for generating new samples is set to 3, with a gamma value of 5 for the radial base kernel similarity function, fine-tuning the model’s sensitivity to minority classes. This comprehensive and detailed experimental setup underscores the approach’s commitment to accuracy and effectiveness in handling complex, imbalanced datasets.

**Table 2:** Parameter settings.

**Parameter**	**Setting**
Number of cross-validation folds	10
Training data percentage	60%
Testing data percentage	20%
Validation data percentage	20%
Kernel functions	Linear; Polynomial (degree 2–5); Sigmoid; radial basis function (RBF)
Number of nearest neighbors to check for a sample in the overlapped region	4
Number of nearest neighbors in the validation set used to evaluate the classification model	10
Percentage of the best classification models used in voting	20%
Number of nearest neighbors of the minority sample used to generate new samples	3
RBF kernel similarity function $\gamma$	5

**Table 3:** Comparison of MSVM-AdaBoost and the proposed kernel-based SVM on all datasets in terms of Accuracy, Precision, and G-Mean (%). Best results for each dataset–metric pair are shown in parentheses.

	**Accuracy**		**Precision**		**G-Mean**	
**Dataset**	MSVM-AdaBoost	Proposed	MSVM-AdaBoost	Proposed	MSVM-AdaBoost	Proposed
ecoli1	(88.44)	86.59	(87.04)	81.28	84.41	(89.28)
ecoli2	88.08	(91.37)	(86.36)	83.53	83.86	(91.76)
ecoli3	87.37	(90.30)	86.41	(89.61)	83.96	(90.37)
ecoli4	92.64	(97.62)	(91.10)	87.17	88.93	(96.25)
glass0	(94.62)	78.23	(92.83)	82.25	(90.19)	80.75
glass2	(93.45)	78.22	(91.89)	78.73	(88.75)	85.85
glass4	(94.88)	84.86	(93.49)	89.56	90.36	(93.99)
glass6	92.61	(95.81)	82.29	(92.09)	82.93	(91.57)
haberman	75.31	(79.65)	74.78	(78.94)	(71.72)	69.71
new-thyroid1	94.82	(94.88)	93.82	(95.62)	90.21	(94.05)
pima	(88.67)	71.48	(87.30)	69.21	(85.22)	68.83
segment0	77.84	(97.83)	75.88	(93.89)	74.18	(97.97)
vehicle0	89.41	(91.95)	88.49	(90.94)	85.14	(93.79)
vehicle1	57.78	(83.84)	56.58	(83.44)	53.97	(83.49)
vehicle3	56.95	(80.81)	55.54	(78.06)	53.05	(79.36)
vowel0	93.74	(97.47)	92.14	(96.05)	89.34	(98.60)
wisconsin	87.64	(96.92)	85.89	(96.56)	83.03	(96.73)
yeast1-7	77.05	(80.39)	75.98	(78.46)	73.44	(77.44)
yeast2-4	66.05	(94.75)	63.73	(84.38)	66.46	(88.65)
yeast3	65.87	(93.46)	64.67	(81.81)	75.19	(91.96)
Avg.	83.16	88.32	77.44	81.51	75.92	83.83
Count (better datasets)	5	15	7	13	4	16

## Results and discussion

**Fig. 7:**
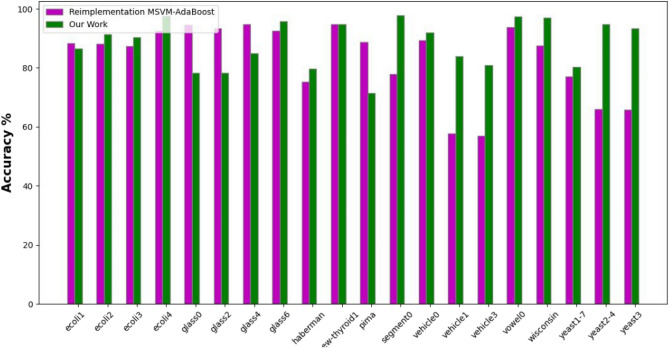
Accuracy.

**Fig. 8:**
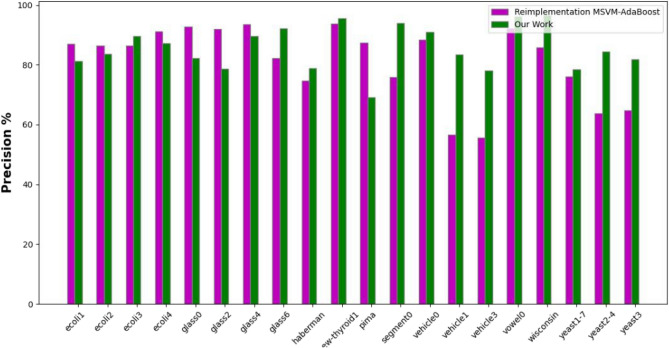
Precision.

**Fig. 9:**
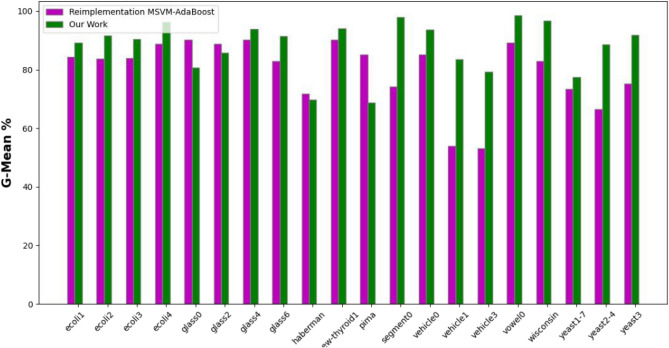
G-mean.

The experimental results, as depicted in the Tables 3–5), present a comprehensive overview of the performance metrics-accuracy, precision, and G-mean-across various datasets for the MSVM, MSVM-AdaBoost, and the proposed kernel-based dynamic ensemble approach (Figs. 7, 8 and 9).

The Precision Table 2 reflects a similar trend, with the proposed approach outperforming others in several datasets, though with a slightly lower average precision. This suggests a balanced approach in classification, managing the trade-off between precision and recall effectively (Table 3).

During the experiment, a dynamic ensemble learning strategy was utilized to tackle the challenge of multi-class imbalance in classification. This approach incorporated a variety of models, each focusing on distinct samples within the overlapping regions. A dynamic model selection process was then applied for classifying test data, based on each model’s effectiveness in classifying validation samples closely related to the test sample. The best-performing models were subsequently employed for majority voting to classify the testing samples.

The comprehensive examination of the experimental results showcases the effectiveness of the proposed kernel-based dynamic ensemble approach in comparison to the established benchmarks by [36]. Across various datasets, the proposed methodology has consistently outperformed or achieved competitive results in key performance metrics compared to MSVM and MSVM-AdaBoost. In terms of accuracy, the proposed approach yields an average of 88.32%, which is a notable improvement over MSVM-AdaBoost’s 83.16%. It also demonstrates supremacy in the majority of the datasets, such as ’ecoli4’, ’segment0’, and ’vowel0’, where it achieved 97.62%, 97.83%, and 97.47% respectively, indicating its robustness in accurately classifying both minority and majority classes. The precision metric, while presenting a slightly lower average for the proposed method (81.51%) compared to MSVM-AdaBoost (77.44%), still shows that the proposed approach holds the lead in the majority of datasets. This suggests that the proposed method can achieve a balanced trade-off between precision and recall, a critical aspect when false positives carry a high cost. In the G-Mean analysis, the proposed approach underscores its balanced sensitivity and specificity by outperforming the other methods in the majority of cases with an average of 83.83%, compared to MSVM-AdaBoost’s 75.92%. This is a critical measure for imbalanced datasets, as it confirms that the proposed method provides a geometric balance in recognizing both minority and majority classes, which is especially significant in medical diagnosis or fraud detection scenarios where the minority class is often the focus. The outcomes highlight the potential of the Kernel-Based Dynamic Ensemble approach to not only advance the current state of machine learning in handling imbalanced datasets but also to address the complexities associated with multi-class datasets that feature overlapping classes. The proposed methodology represents a substantial advancement over the methods explored by Zafar Mehmood et al. (2021). The enhancement in performance metrics across a diverse array of datasets validates the efficacy and adaptability of the proposed approach, paving the way for its application in various real-world scenarios where accurate and equitable classification is paramount. These findings align with the reported superiority of dynamic ensemble learning over AdaBoost, as noted in [2]. Additionally, the results underscore the importance of distinguishing overlapped regions using the kernel with SVM, as indicated in [1].

In the field of machine learning, class imbalance and overlapping decision boundaries pose significant challenges for accurate classification. Real-world applications often involve scenarios where one class is significantly more prevalent than another, leading to biased models. Additionally, overlapping regions in feature space make it difficult to distinguish between classes^9^. Imbalanced learning problems refer to situations where the distribution of classes in the dataset is unequal, leading to an over representation of one class and an under representation of the other. This poses a challenge for classification algorithms as they tend to have lower accuracy for the minority class. This response will provide strategies for handling imbalanced learning problems in binary and multi-class classification tasks based on relevant references.

- **Data Resampling:** It has been demonstrated that data resampling methods such as Synthetic Minority Over-sampling Technique (SMOTE), Borderline-SMOTE, and Adaptive Synthetic Sampling (ADASYN) enhance classification performance in imbalanced datasets.^29^.
- **Algorithm Approaches:** Recent studies have shown that imbalanced datasets can be handled by deep learning algorithms like CNNs and RNNs. Modifications to traditional techniques, such as SVMs and random forests, have also been suggested to alleviate class imbalance.^30^.
- **Cost-Sensitive Learning:** Cost-sensitive learning approaches like Weighted SVM and Adaptive Cost-Sensitive Boosting have been shown to improve the performance of classifiers on imbalanced datasets^31^.
- **Ensemble Methods:** Ensemble methods, such as bagging, Boosting, and Stacking, have improved classification performance on imbalanced datasets^32^.
- **Active Learning:** To address unbalanced datasets, active learning-which entails choosing the most instructive samples for labelling has been suggested. Active learning can enhance classification performance on unbalanced datasets, according to recent research.

### Computational complexity and scalability

- Training Complexity: We explain that the complexity of the training is bigger than that of a single SVM because of the complexity of training the *N* base classifiers and running the process of dynamic selection. We give a complexity analysis, that the training of the SVMs is the most expensive part, which is *O*(*Nn*3)Q where *N* is the number of SVMs trained and n is the number of training samples.
- Inference Complexity: We describe that the inference complexity is small since it only requires to compute the local competence of the top *k* classifiers and a weighted prediction which is very efficient.
- Scalability We observe that the kernel approach is optimized to be scaled to parallel since the framework can be parallelized (base classifier training could be done in parallel) and that future research will need scalability techniques including linear kernels or approximation techniques of very large data sets.

## Conclusion

This paper presents a compelling solution to the intricate challenge of classifying imbalanced datasets with overlapping classes. By systematically addressing the shortcomings of previous models and focusing on the harder-to-classify instances, boosting provides a dynamic and effective approach to dealing with imbalanced datasets.

Through the innovative kernel-based dynamic ensemble approach, we have demonstrated significant strides in achieving superior accuracy and G-mean compared to conventional methods. The robustness exhibited by our approach underscores its immense potential for a myriad of real-world applications, from healthcare to finance and beyond. While celebrating our achievements, we acknowledge the persistent quest for perfection. Precision, in particular, emerges as an area ripe for further exploration. While our approach excels in overall performance, the nuances observed in precision across diverse datasets demand our attention. Thus, we advocate for future research endeavors to delve deeper into refining precision without compromising the integrity of other crucial performance metrics.

## Data Availability

The datasets are available from the corresponding author upon reasonable request.
